# Long read sequencing characterises a novel structural variant, revealing underactive AKR1C1 with overactive AKR1C2 as a possible cause of severe chronic fatigue

**DOI:** 10.1186/s12967-023-04711-5

**Published:** 2023-11-17

**Authors:** Julia Oakley, Martin Hill, Adam Giess, Mélanie Tanguy, Greg Elgar

**Affiliations:** 1 Independent researcher, Hampshire, UK; 2 Department of Steroids and Proteofactors, Institute of Endocrinology, Národni 8, 11694 Prague, Czech Republic; 3 Scientific Research and Development, Genomics England, London, UK

**Keywords:** AKR1C1, AKR1C2, Fatigue, Neurosteroids, Allopregnanolone, ME/CFS diagnosis, Long read sequencing, Structural variants

## Abstract

**Background:**

Causative genetic variants cannot yet be found for many disorders with a clear heritable component, including chronic fatigue disorders like myalgic encephalomyelitis/chronic fatigue syndrome (ME/CFS). These conditions may involve genes in difficult-to-align genomic regions that are refractory to short read approaches. Structural variants in these regions can be particularly hard to detect or define with short reads, yet may account for a significant number of cases. Long read sequencing can overcome these difficulties but so far little data is available regarding the specific analytical challenges inherent in such regions, which need to be taken into account to ensure that variants are correctly identified. Research into chronic fatigue disorders faces the additional challenge that the heterogeneous patient populations likely encompass multiple aetiologies with overlapping symptoms, rather than a single disease entity, such that each individual abnormality may lack statistical significance within a larger sample. Better delineation of patient subgroups is needed to target research and treatment.

**Methods:**

We use nanopore sequencing in a case of unexplained severe fatigue to identify and fully characterise a large inversion in a highly homologous region spanning the *AKR1C* gene locus, which was indicated but could not be resolved by short-read sequencing. We then use GC–MS/MS serum steroid analysis to investigate the functional consequences.

**Results:**

Several commonly used bioinformatics tools are confounded by the homology but a combined approach including visual inspection allows the variant to be accurately resolved. The DNA inversion appears to increase the expression of AKR1C2 while limiting AKR1C1 activity, resulting in a relative increase of inhibitory GABAergic neurosteroids and impaired progesterone metabolism which could suppress neuronal activity and interfere with cellular function in a wide range of tissues.

**Conclusions:**

This study provides an example of how long read sequencing can improve diagnostic yield in research and clinical care, and highlights some of the analytical challenges presented by regions containing tandem arrays of genes. It also proposes a novel gene associated with a novel disease aetiology that may be an underlying cause of complex chronic fatigue. It reveals biomarkers that could now be assessed in a larger cohort, potentially identifying a subset of patients who might respond to treatments suggested by the aetiology.

**Supplementary Information:**

The online version contains supplementary material available at 10.1186/s12967-023-04711-5.

## Background

The dramatic reduction in the cost of high-throughput sequencing over recent years has made short-read whole genome sequencing (WGS) viable as a go-to option for clinical genomic analysis. The high accuracy at base level combined with increasingly sophisticated bioinformatic analytical tools has enabled diagnosis in many cases that were previously unexplained. Despite these advances, however, causative variants cannot be found for up to three quarters of patients thought to have a genetic disorder [1], leaving many still searching for answers.

Disorders of unexplained chronic fatigue (CF), such as myalgic encephalomyelitis/chronic fatigue syndrome (ME/CFS), show strong evidence of heritability with multiple cases in single families, but the inheritance pattern does not appear to be Mendelian [2, 3]. So far no directly causative genes have been discovered [2] and research is hampered by the lack of clear diagnostic biomarkers [2, 3]. The heterogeneous nature of symptoms, triggers and disease progression suggests that the broad diagnostic criteria likely encompass multiple different aetiologies with overlapping symptoms, which may confound any investigation of the population as a whole [2, 3]. These debilitating, multi-system conditions can leave people bedbound for years, unable to speak or feed themselves, often in constant pain and unable to tolerate any light, sound or touch [4]. There is an urgent need to distinguish between subsets of patients in order to better target research and treatment.

There is hope that the rise of next-generation WGS and large-scale genome-wide association studies (GWAS) will bring about a step change in understanding. However, financial constraints mean that GWAS must generally rely on a single nucleotide variant (SNV) array based approach, which by definition cannot identify structural variants (SVs), novel SNVs, or any other SNVs not selected for the array panel. Next-generation WGS may also fall short because some parts of the genome are inherently “dark” to short-read alignment due to high homology or low complexity, where entire reads can map equally well to multiple loci. These regions may be more prone to structural variation and are thought to account for a significant proportion of pathogenic variants [5, 6].

One such region is a stretch of segmental duplication on human chromosome 10p15 to 10p14 that contains the four Aldo–Keto Reductase Family 1 Member C genes. *AKR1C1-4* lie in numerical order, with *AKR1C1* on the forward strand facing *AKR1C2* on the reverse strand then *AKR1C3* and *AKR1C4* both on the forward strand. The four genes share > 86% amino acid sequence identity overall, while *AKR1C1* and *AKR1C2* in particular share nearly 98% amino acid sequence identity and differ by only 7 amino acids [7]. The AKR1C enzymes are well known for their wide-ranging roles in steroid metabolism, including deactivation of progesterone (P) and synthesis of the neuroactive steroids allopregnanolone (allo) and pregnanolone (preg), which positively modulate the gamma-aminobutyric acid type A receptor (GABA_A_R) in a similar way to benzodiazepine sedatives [8]. Progesterone and the GABAergic neurosteroids regulate many different biological functions, especially in the brain [9, 10], suggesting that perturbed metabolism of these steroids could contribute to complex conditions like CF. To date, however, the possible influence of AKR1C enzymes on CF disorders does not appear to have been considered, and germline variants in *AKR1C1* and *1C2* have been surprisingly little studied in any disease context at all [11].

Long read sequencing (LRS) technologies such as those from Oxford Nanopore (ONT) and Pacific Biosciences (PacBio) have the potential to shed light on these dark regions. Long reads are typically in excess of 10 kb in length and can reach several megabases [5], meaning that smaller SVs of up to around 50 kb may be covered in their entirety by single reads, while with larger variants the reads spanning breakpoints are likely to cover enough unique bases to enable accurate alignment, eliminating the multiple-mapping problems of short reads.

Until recently, the relatively low base-level accuracy and high cost of LRS have not permitted clinical adoption, but advances in sequencing chemistry and base calling have now improved accuracy to acceptable levels for the majority of applications [5, 12] and costs continue to fall. Early use of clinical LRS has successfully identified a number of pathogenic variants missed by standard testing, including SVs, complex rearrangements and repeat expansions [5, 6, 12], and mounting evidence suggests that such variants contribute significantly more to genetic disease than previously thought [5, 6, 12]. As yet, however, there are few reports detailing the specific challenges that repetitive regions present for algorithmic analysis or how well the most-used bioinformatic tools perform in practice [5]. A better understanding of where automated pipelines may struggle could increase diagnostic yield by ensuring that fewer variants are missed.

Here, we demonstrate the power of LRS to fully characterise a large inversion in a difficult-to-align region that is all but invisible to short reads. We highlight some of the challenges that can arise when analysing both long read and short read data from such regions, and show that even when SV callers do indicate structural variation they may not correctly resolve the SV, potentially suggesting a functional impact that can differ significantly from the true effect.

We also present biochemical evidence that the variant described here is functional in our patient. We propose that it represents a novel aetiology combining underactive AKR1C1 with overactive AKR1C2, which limits the clearance of intracellular progesterone while suppressing neuronal activity via the GABA_A_R, resulting in a complex, multi-system syndrome of severe fatigue. We further propose that the genetic and biochemical biomarkers discovered in our patient could be assessed in a larger cohort, to see if they constitute a subset of CF patients who might respond to treatments suggested by the aetiology.

## Materials and methods

### Clinical details

#### Presentation

The 42 year old patient has a long and unusually complex history, of which the most debilitating aspect is increasingly severe mental and physical fatigue. Onset occurred after a viral infection age 16, initially mild but gradually progressing. At present, the patient has been completely housebound for many years and is unable to sit up or speak for much of the day.

#### Diagnoses

Clinical diagnoses include central adrenal insufficiency and partial central diabetes insipidus, both thought to be hypothalamic rather than pituitary, along with renal salt wasting and functional pan-enteric dysmotility. There was previously a diagnosis of ME/CFS for some years but that label can be a barrier to getting appropriate treatment and was removed after the later diagnosis of other conditions known to cause persistent fatigue. Mental health assessment concluded that there was no underlying psychiatric disease. None of the current diagnoses, however, fully explains the severity of illness or the full range of symptoms. It is also highly unusual to see this constellation of conditions, especially with young-adult onset. The patient was referred to the UK’s 100,000 Genomes Project (100kGP) on suspicion of Gitelman syndrome due to the clinical presentation of renal salt wasting, but no likely tubulopathy-associated variants have been found and other aspects of her condition are outside the scope of the 100kGP.

#### Atypical steroid response

Of particular note was that certain steroid medications provoked an atypical reaction resembling the effects of benzodiazepines. This inspired a novel hypothesis of excessive neurosteroid-mediated activation of the GABA_A_R, possibly due to upregulation of the metabolic pathways that produce inhibitory GABAergic neurosteroids, which could be either genetic or acquired. The patient reported that a similar but less acute sense of sedation, distinct from the fatigue, had started some years previously. It had originally occurred only in the latter stages of the menstrual cycle but had progressively spread throughout the cycle until it is now constantly present, although still with some cyclic variation.

#### Family history

The family history was suggestive of a genetic predisposition to CF, with unexplained fatigue in several family members (albeit to a much lesser degree), including a period of quite debilitating fatigue in the mother’s early 20s and a maternal second cousin diagnosed with ME/CFS.

#### Expression analysis

RQ-PCR was conducted for a separate study investigating the *AKR1Cs* in relation to a different condition that the patient also has in a mild form, which she joined after the discovery of the inversion in the 100kGP data. The results from a luteal phase sample showed substantial overexpression of *AKR1C2* mRNA in stabilised whole blood, with relative expression approximately 100 times higher than controls, while *AKR1C1* and *AKR1C3* were comparable with controls (full details seen but not reproduced here as the other study has not yet reported).

### Short-read WGS

Whole genome sequence data for the patient and the patient’s mother were provided by the 100kGP. The data were produced via the standard 100kGP pipeline [1] and included the Illumina proprietary workflow of short reads aligned to GRCh38 with small variants called by Starling, plus structural variants called by Manta [13] and Canvas [14]. 97.6% of the genome was covered by at least 15x sequencing depth, with an overall mean of 47.38x.

### Long read WGS

DNA was extracted from 1 mL of whole blood with the Nanobind CBB Big DNA kit (Circulomics NB-900-001-01). After extraction, the genomic DNA (gDNA) was homogenised by 3–10 passes of needle shearing (26G) and 1h of incubation at 50 °C. The gDNA was then split into two aliquots. 4 μg of gDNA were fragmented to ~50kb with the Megaruptor 3 (Diagenode, E07010001 and E07010003) and 10 μg were fragmented to 20-25kb with a gTUBE (Covaris, 520079). All samples were depleted of short DNA fragments (less than 10kb long) with the SRE kit (Circulomics, SS-100-101-01). DNA size distributions were assessed at each step on a FemtoPulse system (Agilent, M5330AA and FP-1002-0275) and all samples were quantified on a Qubit fluorometer (Invitrogen, Q33226).

Two sequencing libraries were generated from 1.5 μg of each gDNA sample with the SQK-LSK109 kit (Oxford Nanopore Technologies). 25 fmol of the obtained sequencing libraries were loaded onto a R9.4 flow cell (ONT) and sequenced on a PromethION48 (ONT). Both flowcells generated approximately 90Gb of mappable data, providing a total sequencing depth of ~60x. The N50s were 23kb and 37kb.

Base-calling was performed with Guppy-4.0.11 using the high accuracy model and a qscore filter of 7. Full details of the protocol are given in Additional file 1. Sequencing reads were aligned to GRCh38 using minimap2 version 2.17 [15]. QC statistics and plots were generated using Nanoplot version 1.26.0 [16]. Structural variants were called with Sniffles version 1.0.11 [17]. Full details of the bioinformatics pipeline are provided in Additional file 2.

### Bioinformatic analysis of the *AKR1C* locus

The variant call files provided by the 100kGP were annotated and filtered using seqr version 0.3.0 [18].

Analysis of the ONT data followed the workflow:

1. Identifying and defining the inversion
2. Phasing
3. Assembling the alleles
4. Annotating the alleles
5. Comparing the long read data to the short reads

Visual inspection in Integrative Genomics Viewer (IGV) [19] was used to identify and define the inversion, and to assess the results of the algorithmic tools at every stage of the analysis. Various elements of Samtools version 1.12 [20] were used to create a custom reference sequence to confirm the inversion and reads were aligned with minimap2 in line with our standard pipeline. Phasing was performed with Samtools Phase. After benchmarking a number of tools, Flye version 2.8.3 [21] was chosen to assemble the alleles with additional polishing by racon v1.4.3 [22]. The assembled alleles were annotated with Liftoff version 1.6.1 [23].

### Serum steroid analysis

The patient provided five samples in total: two from the early follicular phase of the menstrual cycle and three from the mid luteal phase, covering different cycles with symptomatic variation. All samples were taken in the morning. Blood was drawn from the cubital vein, centrifuged at 1790g for 10 min, and the serum stored at −20 °C until analysis.

The samples were prepared and analysed using our previously described GC–MS/MS method [24]. In brief, after addition of mixed internal standards to the sample (1ml), free and conjugated steroids were separated by partition with diethyl-ether. After further preparation, including hydrolysis of the conjugate fraction, each fraction was derivatised first with methoxyamine hydrochloride solution in pyridine then with the reagent Sylon BTZ, then analysed on a Shimadzu GCMS-TQ8040 gas chromatograph-mass spectrometer system fitted with a Restek Rtx-50 capillary column (diameter 0.25 mm, length 15 m, film thickness 0.1 μm) with helium as carrier gas. Analyses were run in multiple reaction monitoring mode using electron-impact ionization with accelerating voltage set at 60 eV and emission current at 151 μA.

82 analytes were reported and concentrations were compared against a pool of controls, who were selected from our records to correspond as closely as possible to the patient. These were healthy, medication-free, premenopausal women, matched to the relevant stage of the menstrual cycle (11 follicular, 19 luteal). The control data were assessed as medians with interquartile range to allow for skewed distributions and non-constant variance.

## Results

### Short-read WGS indicates a large inversion around *AKR1C1* and *AKR1C2*, with a splice-site variant in the 5’UTR of *AKR1C2*

**Fig. 1:**
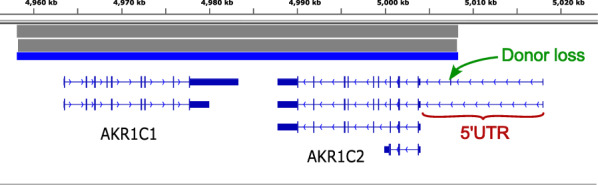
View of the region in IGV, showing the Manta SV calls (grey/blue bars) spanning from approx. 5 kb upstream of *AKR1C1* to part way through the extended 5’UTR of *AKR1C2*, with the splice-donor loss SNV marked just inside the *AKR1C2* end of the inversion calls

The Manta SV calls from the 100kGP indicate a heterozygous large inversion of a region from approximately 5 kb upstream of *AKR1C1* to part way through the extended 5’UTR of the first two transcripts of *AKR1C2* (Fig. 1). This variant seemed potentially relevant since AKR1C1 and AKR1C2 are both instrumental in the synthesis of GABAergic neurosteroids. If the inversion alters protein expression in such a way that AKR1C2 is overexpressed and/or AKR1C1 is partially lost then it would seem to fit precisely with the clinical presentation.

In addition to the inversion call we identified a heterozygous splice-donor loss SNV, NG_031852.1:g.15610G > C (rs67245807), in the second intron of the first transcript of *AKR1C2*, marked with an arrow in Fig. 1. This intron is part of the 5’UTR of *AKR1C2* just inside the inverted region.

Both variants are also present in the patient’s mother, suggesting that they are on the same allele. The splice-site SNV was therefore deemed unlikely to be relevant, since the inversion effectively removes the first exon of the extended 5’UTR meaning that transcript 1 would not be produced. This SNV is also relatively common, with an ALFA Allele Frequency of 0.12712 in Europeans (lower in other populations). It is not listed in ClinVar.

Detailed analysis of the inversion was not possible with the short-read data due to the high homology. In addition, similar large inversion calls were present in a number of unrelated individuals in the 100kGP dataset, so that it was unclear whether the inversion was actually real or merely an alignment artefact caused by the homology. Long read sequencing was therefore sought instead.

### Characterising the variant from the long read data

#### Initial overview

**Fig. 2:**
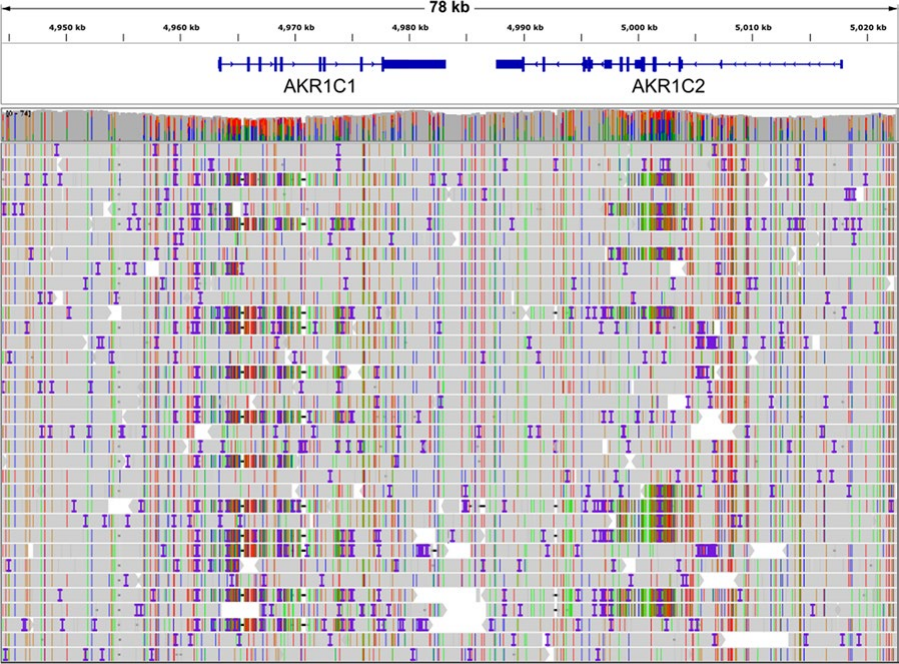
Part of the 60 × coverage Oxford Nanopore sequence viewed in IGV. Multiple reads at each end of the region show a pattern of insertions, deletions and mismatched bases suggestive of a heterozygous structural variant but there are no split reads

SV calling with Sniffles indicates several deep-intronic insertions and deletions (indels) of between 50 and 420 bp in the region of *AKR1C1* and *AKR1C2* but does not detect an inversion. On visual inspection in IGV, however, signs of a heterozygous large SV spanning the region can clearly be seen, although the exact nature of the SV is not immediately obvious (Fig. 2).

After initial phasing with Samtools Phase it can be seen that the REF allele looks normal while the ALT allele has several features suggesting an inversion, including mirrored insertions and deletions where a segment is deleted from one side of the region and the reverse complement of that segment inserted at the equivalent position on the other side. These are the indels that Sniffles had detected. Due to the high homology, however, there is not the classic inversion pattern of reads split into supplementary alignments on opposite strands. Instead, the reads remain intact but with clusters of high-density SNVs interspersed with sections that appear normal, as well as the mirrored indels.

#### Confirming the inversion

Breakpoints were estimated by the loci at which variants in the ONT sequence started to differ significantly from those in the 100kGP sequence. To confirm the inversion we created a new reference sequence consisting of the GRCh38 consensus sequence with the region between the proposed breakpoints inverted. When mapped to this inverted reference the ALT allele fits cleanly while the REF allele now shows the same pattern of variants as the ALT allele had when aligned to the standard reference, but with the pattern reversed (Additional file 3: Fig. S1). The span of the inversion was therefore established as chr10:4,962,657–5,004,621—i.e. 758 bp before exon 1 of *AKR1C1* and 602 bp upstream of the first coding exon of *AKR1C2*.

#### Homology hinders phasing

The phasing itself presents some challenges. The extreme homology means that several reads contained entirely within the inversion cannot be confidently assigned to either allele at the first attempt, then once aligned to the inverted reference it is clear that some of the reads that had been confidently assigned are actually on the wrong allele. The phasing was therefore refined with two further rounds of Samtools Phase on each allele then the final few aberrant reads were moved manually using basic bash commands. This produces two cleanly phased BAM files, one of which closely matches the standard reference while the other closely matches the inverted reference sequence (Additional file 3: Fig. S1).

#### Additional insertion near *AKR1C2*

In addition to the large inversion, the long reads reveal a novel sequence insertion of 310–330 bp on the inverted allele at chr10:4,961,564–4,961,565, 1092 bp upstream of the first breakpoint. It is not possible to determine the precise length due to a homopolymer run at one end, the length of which varies significantly between the reads. This insertion appears to be nsv4544854, which can also be detected visually in the 100kGP data (though it hadn’t been called), but in a slightly different position because repetitive sequence at the end affects short-read alignment.

### Assembling the alignments

Attempts to assemble the ALT allele using Canu and minimap2-miniasm appeared to be confounded by the homology in the region but good results were obtained using Flye, either with 5 rounds of polishing as part of the Flye pipeline or with one round of polishing by Flye followed by one round of polishing with racon. Accuracy was assessed by mapping the raw reads back to the assembly for visual inspection in IGV (Additional file 3: Fig. S2). We then followed the same process to assemble the REF allele for comparison.

**Fig. 3:**
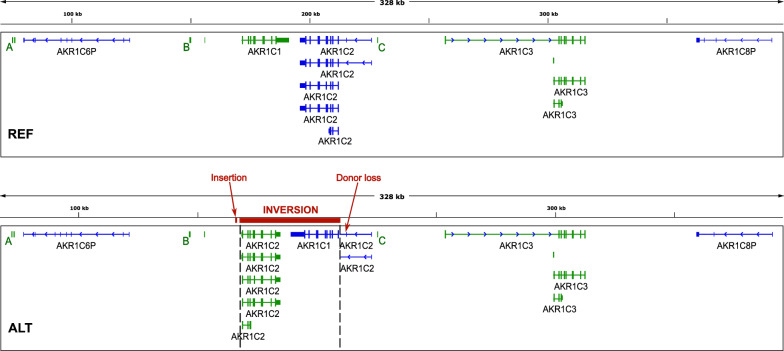
Annotations mapped to the two assembled alleles. Features on the forward strand are coloured green, reverse strand features are blue. Top: REF allele shows the standard arrangement of genes. Bottom: ALT allele shows *AKR1C1* swapped with the coding region of *AKR1C2*. The inversion, the ~320 bp insertion and the donor loss splice-site variant are indicated in red. A, B and C are transcriptional cis-regulatory regions on the forward strand

Liftoff was used to map the RefSeq curated subset of genes and functional element annotations (May 2021) to each of the assembled alleles. As can be seen in Fig. 3, the REF allele shows the expected position and orientation of *AKR1C6P*, *AKR1C1*, *AKR1C2*, *AKR1C3* and *AKR1C8P*, while in the ALT allele *AKR1C1* has been swapped with the coding region of *AKR1C2*.

### Comparison with short-read sequence

**Fig. 4:**
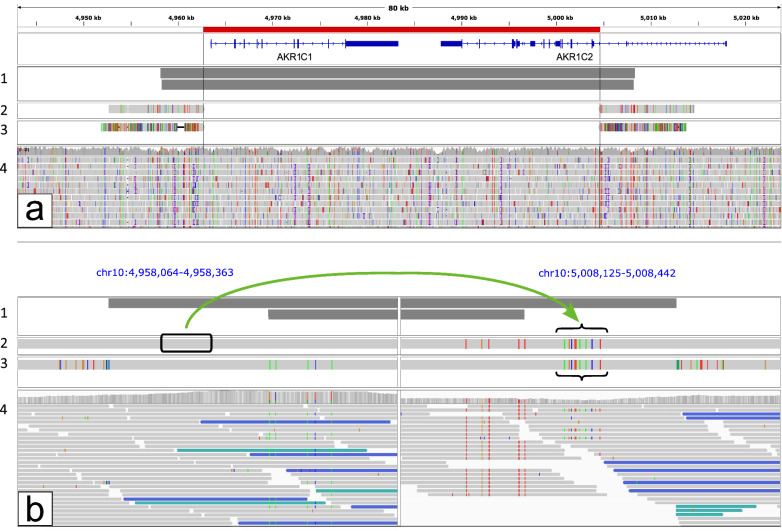
Simulated reads created from the 10 kb of the ALT assembly immediately outside of each breakpoint, shown** a** as an overview, and** b** as a close up (in split screen) at the ends of the Manta inversion calls from the short reads. For both views, 1 is the Manta calls, 2 is the 10 kb simulated reads aligned to the correct sides, 3 is the simulated reads aligned to the opposite sides, and 4 is the short-read sequence with RR- and LL-paired reads shown in turquoise and blue respectively. The green arrow indicates the section that was copied from the *AKR1C1* end and pasted over the equivalent site at the other end, making the two simulated reads identical at that point

The inversion revealed by the ONT sequence is nearly 10 kb shorter than suggested by the short read data. To investigate this discrepancy we created two simulated reads from the 10 kb sections immediately outside of the breakpoints at each end of the ALT assembly. These simulated reads were then aligned back to the GRCh38 reference both in their correct positions and at the opposite sides (Fig. 4a). This enabled a direct comparison of the two sides of the ALT allele, both with each other and with the short reads.

Approximately 5 kb out from the real breakpoints, where Manta had called an inversion due to split and RR/LL oriented reads, there is a short section of 31 bp where the sequence from the *AKR1C1* side appears to have been copied and pasted over the equivalent position on the *AKR1C2* side (Fig. 4b). Both of those loci therefore match the reference at the *AKR1C1* side, which is what had caused the short reads to misalign. All of the apparently split or wrongly oriented read pairs can actually be aligned to GRCh38 in a normal configuration that matches the ALT assembly.

Comparing the simulated reads immediately outside of the breakpoints also revealed why the short-read sequence didn’t detect the real inversion: in the reference sequence the first approx. 570 bases just outside each end of the inversion are almost identical, with only 3 bases different; in the ALT allele those regions are completely identical, with both sides the same as the reference on the *AKR1C1* side. In other words, it appears to be another copy-and-paste. There is then only minimal variation for nearly 300 bp after that.

### Serum steroid analysis suggests a functional effect with increased AKR1C2 and reduced AKR1C1 activity

One of the patient’s two follicular phase samples was from a normal cycle while the other was from what she describes as a “hangover cycle”, where luteal symptoms persist for several days into the follicular phase. This is reflected in the results, with the first having a P concentration comparable with controls at 0.156 nmol/L, while in the “hangover” sample it is much higher at 4.06 nmol/L. Luteal samples also show significant variation in absolute steroid concentrations but the relative ratios between steroids are largely similar across the luteal samples.

Relative concentrations of selected serum steroids (Table 1) were calculated to overcome concentration differences and to reflect the relative activities of AKR1C1 and AKR1C2. Conjugated steroids were high relative to the corresponding unconjugated steroids across the board in the patient compared to controls. We therefore used the sum of free + conjugated steroid, where conjugates were present, as better representing total production. The progesterone-derived AKR1C2 substrates 5α-dihydroprogesterone (5α-DHP) and 5β-dihydroprogesterone (5β-DHP) are not included because their concentrations were below the limit of quantification.

**Table 1:** Steroids for ratios to assess the functional impact of the inversion. Conjugates were included for all steroids except progesterone and 17α-hydroxyprogesterone, which do not undergo significant conjugation

Abbreviation	Steroid	Significance
Allo	Allopregnanolone	Inhibitory neurosteroid
Preg	Pregnanolone	Inhibitory neurosteroid
Iso	Isopregnanolone	Counter-inhibitory neurosteroid
P	Progesterone	Precursor of 20α-reductase (AKR1C1)
17-OHP	17α-Hydroxyprogesterone	Precursor of 20α-reductase (AKR1C1)
20α-DHP	20α-Dihydroprogesterone	20α-reduced metabolite of P
17,20α-diOHP	17α,20α-Dihydroxy-4-pregnen-3-one	20α-reduced metabolite of 17-OHP
DHT	5α-Dihydrotestosterone	Precursor of 3α-reductase (AKR1C2)
Adiol	3α-Androstanediol	3α-reduced metabolite of DHT

**Fig. 5:**
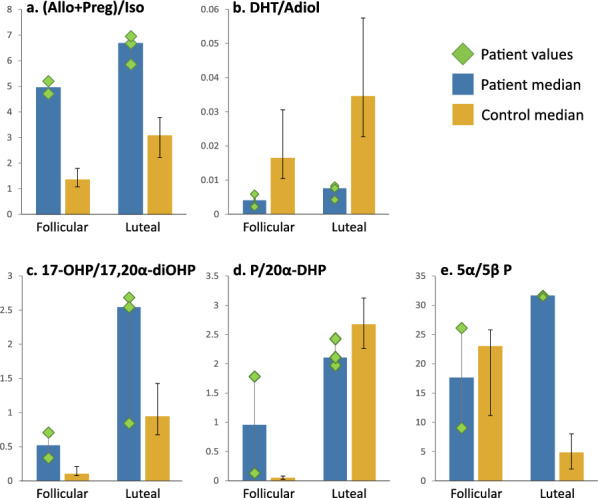
Ratios of steroids indicating: **a** neurosteroid homeostasis; **b** 3α activity of AKR1C2; **c**, **d** 20α activity of AKR1C1; **e** 5α vs 5β reduction of progesterone. Allo: Allopregnanolone; Preg: Pregnanolone; Iso: Isopregnanolone; DHT: 5α-Dihydrotestosterone; Adiol: 3α-Androstanediol; 17-OHP: 17α-Hydroxyprogesterone; 17,20α-diOHP: 17α,20α-Dihydroxy-4-pregnen-3-one; P: Progesterone; 20α-DHP: 20α-Dihydroprogesterone; 5α/5β P: Sum of all 5α- vs 5β-reduced metabolites of progesterone

Results are shown in Fig. 5. The bars represent medians, with results for the patient individually marked and error bars for the controls showing the interquartile range.

#### Neurosteroid homeostasis

The concentration of the main inhibitory neurosteroids, allo and preg, is raised relative to the main counter-inhibitory neurosteroid, iso (Fig. 5a).

#### Precursor-product ratios of AKR1C1 (20α-reduction)

The ratios of 17-OHP/17,20α-diOHP (Fig. 5c) and P/20α-DHP (Fig. 5d) were used to assess the 20α-reduction activity of AKR1C1. 17-OHP/17,20α-diOHP is raised, except in one luteal sample. This sample contained a relatively high cortisol concentration due to higher adrenocortical stimulation, which would have resulted in increased utilisation of 17-OHP to produce cortisol, leaving less available for 20α-reduction (explained further in Additional file 4). P/20α-DHP is raised in the follicular phase but normal or slightly low in the luteal phase.

To see if the normal-low luteal P/20α-DHP ratio could be due to excess P being diverted down the 5α pathway instead of the 20α pathway we examined the sum of all 5α progesterone metabolites versus the sum of all 5β progesterone metabolites, as an indication of relative 5α-reductase (5α-R) activity (Fig. 5e). The 5α/5β ratio for progesterone metabolites (5α/5β P) is high in the luteal phase.

#### Precursor-product ratio of AKR1C2 (3α-reduction)

The ratio of DHT/Adiol, an indicator of 3α-reduction by AKR1C2, is low (Fig. 5b).

## Discussion

Long-read sequencing has not yet filtered through to routine clinical practice, partly due to the high cost and analytical challenges. Variants in difficult-to-align regions may not show the same patterns, such as split or supplemental reads, that would be seen with the same variant in a more straightforward part of the genome. The different patterns may cause SVs to be missed by some variant callers, and while algorithmic tools are rapidly improving they can still struggle, potentially introducing errors that would hinder the downstream analysis or affect interpretation of the variant. In this case we found that despite high coverage, phasing was only partly successful due to the homology; several rounds of Samtools Phase and additional manual manipulation were required to define fully phased alleles that could then be assembled. Even with clean phasing, the homology still confounded some commonly used assemblers. Despite the challenges, however, long-read sequencing can already offer real hope for desperate patients by illuminating regions where short reads fail.

In our case the original short-read sequence did indicate an inversion but what it had picked up was, in fact, an alignment artefact due to a small copy-and-paste error some 5 kb further out. The true inversion is effectively invisible to short reads because any reads spanning the breakpoints would align directly to the other end, while only those pairs with the longest inserts would reach from inside the inversion to the more divergent region further out and so be correctly aligned to opposite ends. In the patient’s short-read sequence there are two such correctly aligned pairs, in the mother's there are none at all. Of course, that also means there's no way of knowing how common this inversion is, since it would not be detected in any of the short-read-based datasets currently available.

The narrower span of the true inversion significantly alters interpretation of the splice-donor loss SNV, which is now seen to be outside of the inversion and therefore part of the extra 5’UTR attached to *AKR1C1*, possibly introducing a retained intron immediately upstream of the translation start site. The narrower span also makes it more likely that the inversion is functional, since the breakpoints are much closer to the coding regions of both genes leaving more of the regulatory regions outside.

*AKR1C2* has 4 coding transcript variants in the Consensus Coding Sequence (CCDS): transcripts 1 & 2 (CCDS7062) differ only in their 5’UTR and both encode the full AKR1C2 protein of 323 amino acids (isoform 1). Transcript 3 (CCDS44350) encodes isoform 2, a truncated version of only 139 amino acids that is predicted to be inactive 7. Transcript 4 (CCDS81438) lacks the extended 5’UTR of transcripts 1 & 2 and is also missing one exon from the coding region; it encodes the 297 amino acid isoform 3. The RefSeq curated subset also includes a fifth transcript variant, which encodes the full isoform 1 of transcripts 1 & 2 but without the extended 5’UTR. *AKR1C1* has only one transcript in the CCDS.

**Fig. 6:**
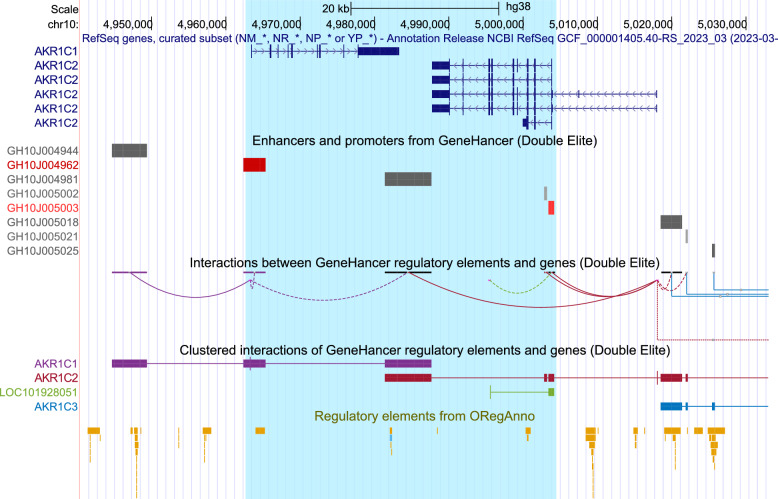
UCSC Genome Browser view of regulatory elements and their interactions in the region, with the inversion highlighted in blue (http://genome.ucsc.edu) [26]

The high homology on opposite strands gives this inversion rotational near-symmetry and effectively swaps *AKR1C1* with the coding region of *AKR1C2*. This has a number of consequences:

1. The initial non-coding exons of *AKR1C2* transcripts 1 & 2 are still in their correct place, meaning that transcription could presumably be initiated in the normal way and so potentially result in a rearranged *AKR1C1* mRNA with those additional exons and possible retained intron added at the 5’ end. It seems likely that this would affect expression and/or translation of *AKR1C1*.
2. Without those first non-coding exons, *AKR1C2* would only have transcript variants 3, 4 and 5. Transcripts 4 and 5 would presumably still produce a functional protein but it seems likely that expression would be altered.
3. The orientation and positions of *AKR1C1* and *1C2* are reversed relative to *AKR1C6P*, *AKR1C3*, *AKR1C8P*, and to all of the regulatory regions outside of the breakpoints. These include three transcriptional cis-regulatory regions on the forward strand in the RefSeq curated subset, marked A, B and C in Fig. 3, that have been shown to positively regulate *AKR1C1* with high confidence in K562 cells. The middle of these (B) also positively regulates *AKR1C2*. GeneHancer indicates other regulatory elements with interactions potentially disrupted by the inversion, as does the ORegAnno database (both shown in Fig. 6), and the MoLoTool sequence motif location tool [25] used with the assembled alleles suggests several candidate steroid hormone receptor binding sites close to the breakpoints.

Expression of the different AKR1C proteins and isoforms is tissue-specific, with AKR1C4 largely confined to the liver while 1C1-3 are expressed in multiple tissues with distinct distribution patterns [7, 27]. The main roles of the AKR1Cs include regulation of active and inactive intracellular steroid hormone concentrations, prostaglandin metabolism, synthesis and binding of bile acids, and as Phase I metabolising enzymes including as part of the antioxidant defence system [7]. All of the AKR1C enzymes can accept a wide range of substrates and exhibit 3-, 17- and 20-ketosteroid reductase activity, but with different preferences for substrate, stereochemistry and reduction position [7].

AKR1C2 preferentially acts as a 3α-reductase, and is the primary enzyme for synthesis of the inhibitory GABAergic neurosteroids allo, preg and allotetrahydrodeoxycorticosterone (THDOC) from 5α-DHP, 5β-DHP and 5α-dihydrodeoxycorticosterone (DHDOC) respectively [7, 28]. Allo and preg in particular are potent positive modulators of the GABA_A_R, making the receptor more responsive to circulating GABA, which in turn suppresses signalling in both the central nervous system and peripheral tissues and so has wide-ranging effects, particularly in the brain [10, 29, 30].

AKR1C1 acts predominantly as a 20α-reductase (20α-R) that reduces active steroids to their inactive metabolites, particularly P to 20α-DHP. AKR1C1 is also thought to be the main enzyme producing the 3β metabolites of 5α-reduced steroids [31]; these include iso, which counters the effects of allo and preg at the GABA_A_R [10, 30].

**Fig. 7:**
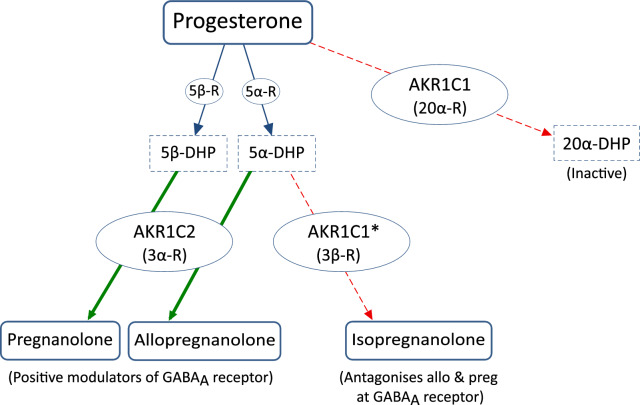
Simplified overview of synthesis of the major GABAergic neurosteroids allopregnanolone, pregnanolone and isopregnanolone. Thick green arrows indicate the pathways upregulated by the inversion, while thin, dashed, red arrows indicate pathways that may be partly inhibited. 5α-R: 5α-reductase; 5β-R: 5β-reductase; 5α-DHP: 5α-dihydroprogesterone; 5β-DHP: 5β-dihydroprogesterone; 20α-R: 20α-reductase; 20α-DHP: 20α-dihydroprogesterone; 3α-R: 3α-reductase; 3β-R: 3β-reductase. *Enzyme predicted in silico to be AKR1C1 but not yet tested in vitro [31, T. Penning, personal communication, 2023]

If the inversion suppresses AKR1C1 while upregulating AKR1C2 then these properties could fit well with the clinical presentation. As shown in Fig. 7, if AKR1C1 is underactive and AKR1C2 overactive then more progesterone will likely be available for the 5α- and 5β-reduction pathways instead of being deactivated to 20α-DHP, and will then be 3α-reduced to allo and preg rather than 3β-reduced to iso, disrupting neurosteroid homeostasis.

The overlapping activities of the AKR1Cs and the complex pathways of steroid metabolism more widely can make it challenging to assess individual enzymes from peripheral blood. Hepatic AKR1C4 in particular has high catalytic efficiency for both 20α- and 3α- reduction [7] and would likely compensate for either excess or deficiency in the other enzymes. Furthermore, it is known that locally synthesised steroids, e.g. in the brain, may reach functionally significant levels without any corresponding variation in peripheral blood [10]. However, neurosteroids can cross the blood–brain barrier so that fluctuations in circulating concentrations, e.g. due to the menstrual cycle, can be reflected in the brain [32, 33], and serum results may still indicate a general tendency that could potentially be more pronounced in other tissues.

The high ratio of (allo + preg)/iso directly supports the hypothesis of a relative increase in inhibitory GABAergic neurosteroids in our patient, with the higher value in the luteal phase corresponding to increased symptoms in the latter part of the menstrual cycle.

The high ratios of 17-OHP/17,20α-diOHP and follicular P/20α-DHP are consistent with partial loss of AKR1C1. The normal-low luteal P/20α-DHP ratio is surprising but could be explained by compensatory diversion of P down the 5α pathway.

5α-R is ubiquitous while the majority of 5β-reduction occurs in the liver [34], meaning that the 5α/5β ratio provides a measure of where 5-reduction is occurring as well as indicating possible alterations in enzyme capacity. The high 5α/5β ratio for P metabolites in the luteal phase suggests a shift to extrahepatic 5-reduction of P. Taken together, the P/20α-DHP and 5α/5β ratios suggest that intracellular P may be accumulating in extrahepatic tissues due to limited 20α-reduction by AKR1C1 and is then being metabolised by 5α-R instead.

The low ratio of DHT/Adiol is consistent with increased 3α-reduction by AKR1C2, with the caveat that adiol can also be synthesised from allo, via androsterone, bypassing DHT and potentially altering the DHT/Adiol ratio. The overexpressed *AKR1C2* mRNA in luteal phase also indicates that AKR1C2 activity may be increased.

All together, these results support the hypothesis of excessive neurosteroid-mediated activation of the GABA_A_R suggested by the clinical presentation, and indicate that this is due to overactive AKR1C2 combined with partial loss of AKR1C1. It seems possible that for *AKR1C2* the inversion either creates an enhancer or removes a silencer. RQ-PCR did not find low *AKR1C1* mRNA expression, as might be expected with the underactive enzyme, but expression could be tissue-specific. It is also possible that gene expression is normal but the extra 5’UTR exons, with possible retained intron, interfere with translation and so cause loss of protein expression or function on that allele.

If this variant is indeed causing overactivity of AKR1C2 while limiting AKR1C1 then it could be part of the underlying cause not only of the fatigue and sedation, due to the sedative effects of allo and preg, but also of the endocrine, renal and gastrointestinal dysfunction in this patient. Excessive GABAergic inhibition in the hypothalamus could suppress the release of corticotropin releasing hormone (CRH) and antidiuretic hormone (ADH), resulting in cortisol deficiency with diabetes insipidus [35, 36]. AKR1C1 (but not 1C2) is highly expressed in the proximal tubule of the kidney [37, 38], where impaired progesterone clearance could interfere with sodium reabsorption through inhibition of the mineralocorticoid receptor and other electrolyte-regulating proteins, resulting in renal salt wasting [39, 40]. AKR1C1, 1C2 and the GABA_A_R are all active in the gastrointestinal tract, where GABA is involved in regulating secretion and motility [37, 38, 41].

More broadly, variants in *AKR1C1* and *1C2* could potentially affect any tissue where steroid hormones undergo 20α- or 3,5-reduction, with implications for a variety of conditions where the aetiology is unknown or a subset of cases remains unexplained. This would be particularly the case for multi-system chronic fatigue disorders like ME/CFS, fibromyalgia and long covid. Gastrointestinal dysfunction and hypocortisolism are common in these conditions, as is dysregulation of the autonomic nervous system [42–44] which may similarly be modulated by neurosteroids [45, 46]. These conditions are also more common in women [47, 48], as might be expected if higher P is a factor. If there is variable expressivity then such variants could also contribute to more tissue- or organ-specific disorders where increased GABA activity or impaired progesterone clearance could play a role, such as irritable bowel syndrome or Gitelman-like renal salt wasting, or to psychiatric disorders that may be related to increased allo activity in the brain such as premenstrual dysphoric disorder [10].

We therefore plan further investigations, including RNA-seq to ascertain whether or not the variant *AKR1C1* mRNA has the extra 5’UTR exons and retained intron, and to assess the relative expression of the two genes and their isoforms. In vitro functional studies would permit direct investigation of the effect of the inversion on protein expression and function, and further metabolomic analysis, if possible, could assess analytes that would give a more direct indication of AKR1C2 activity in neurosteroid synthesis but which could not be accurately measured with GC–MS/MS, such as 5α-DHP, 5β-DHP, DHDOC and THDOC. We also hope to extend the study to other patients, ideally in collaboration with large-scale projects such as DecodeME [49], to investigate whether variants in *AKR1C1* & *1C2* and consequent alterations in steroid metabolism are associated with disease in a subset of patients.

If this hypothesis is correct then it also raises the possibility of treatment. The commonly used and well tolerated bile acid ursodeoxycholic acid (UDCA) is a selective AKR1C2 inhibitor that can penetrate the brain [50, 51], and so may address the root cause of excessive inhibitory neurosteroid synthesis, while bioidentical iso (Sepranolone) and the GABA_A_R modulating steroid antagonist GR3027 (Golexanolone) are showing promise in clinical trials for a variety of conditions associated with relative allo excess [10].

This study has some limitations. As a single case without functional studies it cannot demonstrate conclusively that the inversion is the direct cause of the biochemical abnormalities, or that those abnormalities do indeed underlie the patient’s symptoms. The relatively small number of controls, combined with the high inter- and intra-individual variability of steroid hormone concentrations, means that the control set may not fully represent the population as a whole. Furthermore, while those controls were selected to be as closely matched to the patient as possible, it would have been preferable to recruit volunteers specifically for this study had resources allowed.

It seems extremely fortunate that in this case there was an error that could be detected by the short-read alignment, since this inversion would otherwise have been missed. Perhaps it was all part of the same event, with the DNA break being repaired by gene conversion but with the homology leading to the central section being fully inverted with the copy-and-paste errors further out. With such high homology making errors more likely, however, it is impossible to be sure that it was not sheer coincidence.

## Conclusions

1. Long-read sequencing has the power to identify and fully characterise structural variants in difficult-to-align regions, although the challenges that these regions present to algorithmic analysis mean that manual oversight of the bioinformatic pipeline is currently needed to ensure that variants are accurately identified. In particular, reads crossing SV breakpoints may be aligned intact rather than split, instead showing clusters of SNVs and/or mid-scale indels that may confound SV callers but are easily seen visually. Improved detection of variants in these regions has the potential to provide a molecular diagnosis for more of the many patients with clinically diagnosed genetic disorders, and to reveal the underlying causes of conditions of unknown aetiology. We hope that as costs fall and algorithms improve, long-read methodologies will be widely adopted in clinical practice and research.
2. Genetic variants causing upregulation of AKR1C2 combined with partial loss of AKR1C1 may constitute a novel pathology that underlies some cases of unexplained chronic fatigue. If they do then the biomarkers discovered in this study might allow better targeting of a specific patient subgroup for research and treatment. Genetic screening could be implemented, a serum steroid profile that reports the relevant ratios could be established as a simple diagnostic test, and clinical trials of UDCA, Sepranolone or Golexanolone could be conducted. Follow up studies with transcriptional and functional analyses of the inversion are recommended, along with genetic and biochemical assessment of other patients to determine if this is the case.

### Supplementary Information

**Additional file 1.** Genomic DNA by Ligation protocol.

**Additional file 2.** PromethION SV calling pipeline.

**Additional file 3: Fig. S1.** The cleanly phased BAM files aligned to consensus and inverted reference sequences. In both panels, the upper alignments track is the ALT allele and the lower track is the REF allele. The inverted region is shown by the red bar. In panel **a** the alleles are aligned to the GRCh38 consensus sequence; the ALT allele shows signs of a large inversion while the REF allele fits well. In panel **b** the reads are aligned to the new reference, where the region between the proposed breakpoints is inverted; now the ALT allele fits well while the REF allele shows the signs of a large inversion. **Fig. S2.** The raw reads from the phased ALT allele mapped to the assembled ALT allele. INDELs of ≤ 5 bp are hidden. There are very few mismatched bases and no loci where more than half of the reads have a mismatched base, showing that the assembly is accurate.

**Additional file 4:** Further explanation of the lower 17-OHP/17,20α-diOHP ratio in the patient’s first luteal sample.

## Data Availability

Details of the large inversion are deposited in the European Variation Archive (EVA) with accession number ERZ21343151. The datasets generated during the current study are not publicly available to protect the privacy of the patient but are available from the corresponding author on reasonable request.

## Abbreviations

100kGP: 100,000 Genomes Project
17-OHP: 17α-Hydroxyprogesterone
17,20α-diOHP: 17α,20α-Dihydroxy-4-pregnen-3-one
20α-DHP: 20α-Dihydroprogesterone
ADH: Antidiuretic hormone
Adiol: 3α-Androstanediol
Allo: Allopregnanolone
CCDS: Consensus coding sequence
CF: Chronic fatigue
CRH: Corticotropin releasing hormone
DHDOC: 5α-Dihydrodeoxycorticosterone
DHT: 5α-Dihydrotestosterone
GABA_A_R: Gamma-aminobutyric acid type A receptor
gDNA: Genomic DNA
GWAS: Genome-wide association studies
IGV: Integrative Genomics Viewer
Indels: Insertions and/or deletions
Iso: Isopregnanolone
LRS: Long read sequencing
ME/CFS: Myalgic encephalomyelitis/chronic fatigue syndrome
ONT: Oxford Nanopore Technologies
P: Progesterone
PacBio: Pacific biosciences
Preg: Pregnanolone
SNV: Single nucleotide variant
SV: Structural variant
THDOC: 5α-Tetrahydrodeoxycorticosterone
UDCA: Ursodeoxycholic acid
WGS: Whole genome sequencing
