# Supplementary material for: Long read sequencing characterises a novel structural variant, revealing underactive AKR1C1 with overactive AKR1C2 as a possible cause of severe chronic fatigue
Source: J Transl Med. 2023 Nov 17;21:825. doi: 10.1186/s12967-023-04711-5 (PMC10655400; doi:10.1186/s12967-023-04711-5)
Supplement: Supplementary file 1 — Additional file 1. Genomic DNA by Ligation protocol. [file 12967_2023_4711_MOESM1_ESM.pdf]

v1_protocol_ONT_LSK109 (09/07/2020)

This is a modified version of Oxford Nanopore Technologies protocol SQK-LSK109 "Genomic DNA by Ligation" protocol, Version: GDE_9063_v109_revS_14Aug2019. Please refer to the manufacturer protocol for additional instructions regarding consumables and reagents (https://store.nanoporetech.com/uk/media/wysiwyg/pdfs/SQK-LSK109/Genomic_DNA_by_Ligation_SQK-LSK109_-minion.pdf). Use wide bore pipette tips at all times when pipetting DNA samples.

1. Prepare the DNA in nuclease-free water.
   - Transfer 0.5-1.5 µg of genomic DNA into a DNA low retention tube and adjust the volume to 48 µl with nuclease-free water.
   - Mix thoroughly by inversion avoiding unwanted shearing and spin down briefly in a microfuge.

2. DNA End-Repair and dA-tailing
   - In a DNA low retention tube prepare the following mix:

Reagents	Volume
gDNA	48 µl
NEBNext FFPE DNA Repair Buffer (M6630)	3.5 µl
NEBNext FFPE DNA Repair Mix (M6630)	2 µl
Ultra II End-prep reaction buffer (E7546)	3.5 µl
Ultra II End-prep enzyme mix (E7546)	3 µl
Total	60 µl

   - Mix gently by flicking the tube, spin down and incubate for 10 minutes at room temperature (RT) and for 10 minutes at 65° C. In the meantime, prepare 500 µl of 70% Ethanol per sample.
   - Transfer the DNA sample to a clean 1.5 ml Eppendorf DNA low retention tube.
   - Add 60 µl of re-suspended Agencourt AMPure XP beads to the end-prep reaction and mix by flicking the tube.
   - Incubate on a rotator mixer at 12 rpm for 10 minutes at RT.
   - Spin down the sample and pellet on a magnet. Keep the tube on the magnet, and pipette off the supernatant.
   - Keep on magnet, wash beads with 200 µl of freshly prepared 70% ethanol without disturbing the pellet. Remove the 70% ethanol using a pipette and discard.

- Repeat the previous step.
- Spin down and place the tube back on the magnet. Pipette off any residual ethanol. Allow to dry for ~30 seconds, but do not dry the pellet to the point of cracking.
- Remove the tube from the magnetic rack and re-suspend the pellet in 66 µl nuclease-free water. Incubate for 2 minutes at RT (5 minutes if un-fragmented DNA).
- Pellet the beads on a magnet until the eluate is clear and colourless.
- Remove and retain 64.5 µl of eluate into a clean 1.5 ml Eppendorf DNA low retention tube.

STOP POINT: it is possible to store the DNA at 4°C overnight or proceed immediately to step 5.

3. Adapter ligation and clean-up.
   - Spin down Adapter Mix (AMX) and T4 Ligase from the NEBNext Quick Ligation Module (E6056), and place on ice.
   - Thaw Ligation Buffer (LNB) at RT, spin down and mix by pipetting. Due to viscosity, vortexing this buffer is ineffective. Place on ice immediately after thawing and mixing.
   - Thaw the Elution Buffer (EB) at RT, mix by vortexing, spin down and place on ice.
   - In a 1.5 ml DNA low retention Eppendorf tube prepare the following mix:

Reagents	Volume
DNA	64.5 µl
Ligation Buffer (LNB)	25 µl
NEBNext Quick T4 DNA Ligase (E6056)	8 µl
Adapter Mix (AMX)	2.5 µl
Total	100 µl

   - Mix gently by flicking the tube, spin down and incubate at RT for 30 minutes.
   - Add 40 µl of re-suspended AMPure XP beads to the end-prep reaction and mix by flicking the tube.
   - Incubate on a rotator mixer at 12 rpm for 10 minutes at RT. In the meantime, thaw Long Fragment Buffer (LFB) at RT.
   - Spin down the sample and pellet on a magnet. Keep the tube on the magnet, and pipette off the supernatant.
   - Wash beads with 250 µl of Long Fragment Buffer (LFB). Flick the beads to re-suspend, then return the tube to the magnetic rack and allow the beads to pellet. Remove the supernatant using a pipette and discard.
   - Repeat the previous step.

- Spin down and place the tube back on the magnet. Pipette off any residual buffer. Allow to dry for ~30 seconds, but do not dry the pellet to the point of cracking.
- Remove the tube from the magnetic rack and re-suspend the pellet in 26 µl of Elution buffer (EB). Incubate for 15 minutes at 37°C (This can help recover long DNA fragments).
- Pellet the beads on a magnet until the eluate is clear and colourless.
- Remove and retain 25 µl of eluate into a clean 1.5 ml Eppendorf DNA low retention tube.
- Quantify 1 µl of eluted sample using a fluorometer.
- Check DNA average size (optional – e.g. with Agilent FEMTO Pulse) and prepare 25 fmol for loading onto the flow cell (a minimum of 5 fmol and a maximum of 50 fmol can be loaded per flow cell). If starting from un-fragmented HMW DNA, use 300ng per flow-cell. Store in ice until ready. If necessary, dilute the DNA for a final volume of 24 µl.

4. Priming and loading the flow cells.
    - Thaw the Sequencing Buffer (SQB), Loading Beads (LB), Flush Tether (FLT) and one tube of PromethION Flush Buffer (FB) at room temperature before placing the tubes on ice as soon as thawing is complete.
    - Mix the Sequencing Buffer (SQB) and PromethION Flush Buffer (FB) tubes by vortexing, spin down and return to ice.
    - Spin down the Flush Tether (FLT) tube, mix by pipetting, and return to ice.
    - Prepare the flow cell priming mix: add 30 µl of thawed and mixed Flush Tether (FLT) directly to the tube of thawed and mixed PromethION Flush Buffer (FB); mix by pipetting up and down.
    - Load the flow cell(s) into the docking ports within the PromethION. See video at https://community.nanoporetech.com/protocols/1d-gDNA-sqk-lsk109-prom/v/gde_9077_v109_revh_04jun2018/option-2-priming-and-load).
    - Turn the valve to expose the inlet port
    - After opening the valve, a small tract of air will be visible beyond the inlet port. With a P1000 set to 200 µl, draw back a small volume to remove any air bubbles (a few µls), until you can see a small volume of buffer entering the pipette tip.
    - Using a P1000 pipette, load 500 µl of the Priming Mix flush into the inlet port of the flow cell, avoiding the introduction of air bubbles
    - Wait five minutes. During this time, you can prepare your library for loading, as described in the next steps.
    - Repeat the priming step with another 500 µl flush

- Thoroughly mix the contents of the SQB and LB tubes by pipetting.
- In a 1.5 ml low retention Eppendorf tube prepare the following mix:

Reagents	Volume
SQB	75 µl
LB	51 µl
DNA Library	24 µl
Total	150 µl

- Load 150 µl of your sample through the inlet port.
- Close the valve to seal the inlet port.
- If no further flow cells are to be loaded, close the PromethION lid.
- Wait a minimum of 10 minutes, and up to 3 hours, after loading the flow cells into the PromethION before initiating any experiments. This will help to increase the sequencing throughput.
