# Supplementary material for: Long read sequencing characterises a novel structural variant, revealing underactive AKR1C1 with overactive AKR1C2 as a possible cause of severe chronic fatigue
Source: J Transl Med. 2023 Nov 17;21:825. doi: 10.1186/s12967-023-04711-5 (PMC10655400; doi:10.1186/s12967-023-04711-5)
Supplement: Supplementary file 2 — Additional file 2. PromethION SV calling pipeline. [file 12967_2023_4711_MOESM2_ESM.pdf]

PromethION SV calling pipeline

Version 1.0
28/05/2020

Structural variant sets for comparison:

Audano common SVs - A set of 9046 structural variants common to most human populations. I defined this set as all structural variants occurring in >= 10 of the 15 samples in the study: https://www.ncbi.nlm.nih.gov/pubmed/30661756

Reference genomes:

GRCh38:
ftp://ftp.ncbi.nlm.nih.gov/genomes/all/GCA/000/001/405/GCA_000001405.15_GRCh38/seqs_for_alignment_pipelines.ucsc_ids/GCA_000001405.15_GRCh38_no_alt_analysis_set.fna.gz

Pipeline overview:

1) Merge fastq
2) Map to genome
3) QC
4) Call structural variants
5) Comparison of structural variants to benchmark set

Running the SV pipeline:

Merge fastq:

```
cat ${run}/fastq_pass/*.fastq.gz >
${run}/${sample_id}_fastq_combined.fastq.gz
```

Map to genome:

Minimap2 version 2.17

```
minimap2 --secondary=no --MD -t <threads> -a $genome_index
${run}/${sample_id}_fastq_combined.fastq.gz | samtools sort -o
${out_dir}/${sample_id}_mapped.bam
```

QC stats and plots:

Nanoplot version 1.26.0

```
NanoPlot --raw --store --N50 --prefix $sample_id -t $threads -o
${run}/nanoplot_fastq/ -p fastq --fastq
${run}/${sample_id}_fastq_combined.fastq.gz

NanoPlot --raw --store --N50 --prefix $sample_id --percentqual --alength -t
$threads -o ${run}/nanoplot_aligned_no_alt/ -p aligned --bam
${run}/aligned_minimap_no_alt/${sample_id}_mapped.bam
```

Call structural variants:

Sniffles version 1.0.11

```
sniffles -t $threads --min_support 3 --minmapping_qual 20 --min_seq_size
1000 --report_read_strands --genotype -m
${run}/aligned_minimap_no_alt/${sample_id}_mapped.bam -v
${out_dir}/${sample_id}_mapped_sniffles_robust.vcf
```

Compare structural variants to benchmarking set

Truvari version 1.2

```
#sort
bcftools sort ${in_dir}/${sample_id}_mapped_sniffles.vcf >
${in_dir}/${sample_id}_mapped_sniffles_robust_sorted.vcf

#compress
bgzip ${in_dir}/${sample_id}_mapped_sniffles_robust_sorted.vcf

#index
tabix ${in_dir}/${sample_id}_mapped_sniffles_robust_sorted.vcf.gz

#compare to SV "truth" set
truvari --noprog --pctsize 0 --pctsim 0 -t -c
${in_dir}/${sample_id}_mapped_sniffles_robust_sorted.vcf.gz -b
$common_SV_set -o $out_dir
```
