# Supplementary material for: Long read sequencing characterises a novel structural variant, revealing underactive AKR1C1 with overactive AKR1C2 as a possible cause of severe chronic fatigue
Source: J Transl Med. 2023 Nov 17;21:825. doi: 10.1186/s12967-023-04711-5 (PMC10655400; doi:10.1186/s12967-023-04711-5)
Supplement: Supplementary file 3 — Additional file 3: Fig. S1. The cleanly phased BAM files aligned to consensus and inverted reference sequences. In both panels, the upper alignments track is the ALT allele and the lower track is the REF allele. The inverted region is shown by the red bar. In panel a the alleles are aligned to the GRCh38 consensus sequence; the ALT allele shows signs of a large inversion while the REF allele fits well. In panel b the reads are aligned to the new reference, where the region between the proposed breakpoints is inverted; now the ALT allele fits well while the REF allele shows the signs of a large inversion. Fig. S2. The raw reads from the phased ALT allele mapped to the assembled ALT allele. INDELs of ≤ 5 bp are hidden. There are very few mismatched bases and no loci where more than half of the reads have a mismatched base, showing that the assembly is accurate. [file 12967_2023_4711_MOESM3_ESM.pdf]

The cleanly phased alleles aligned to consensus and inverted references

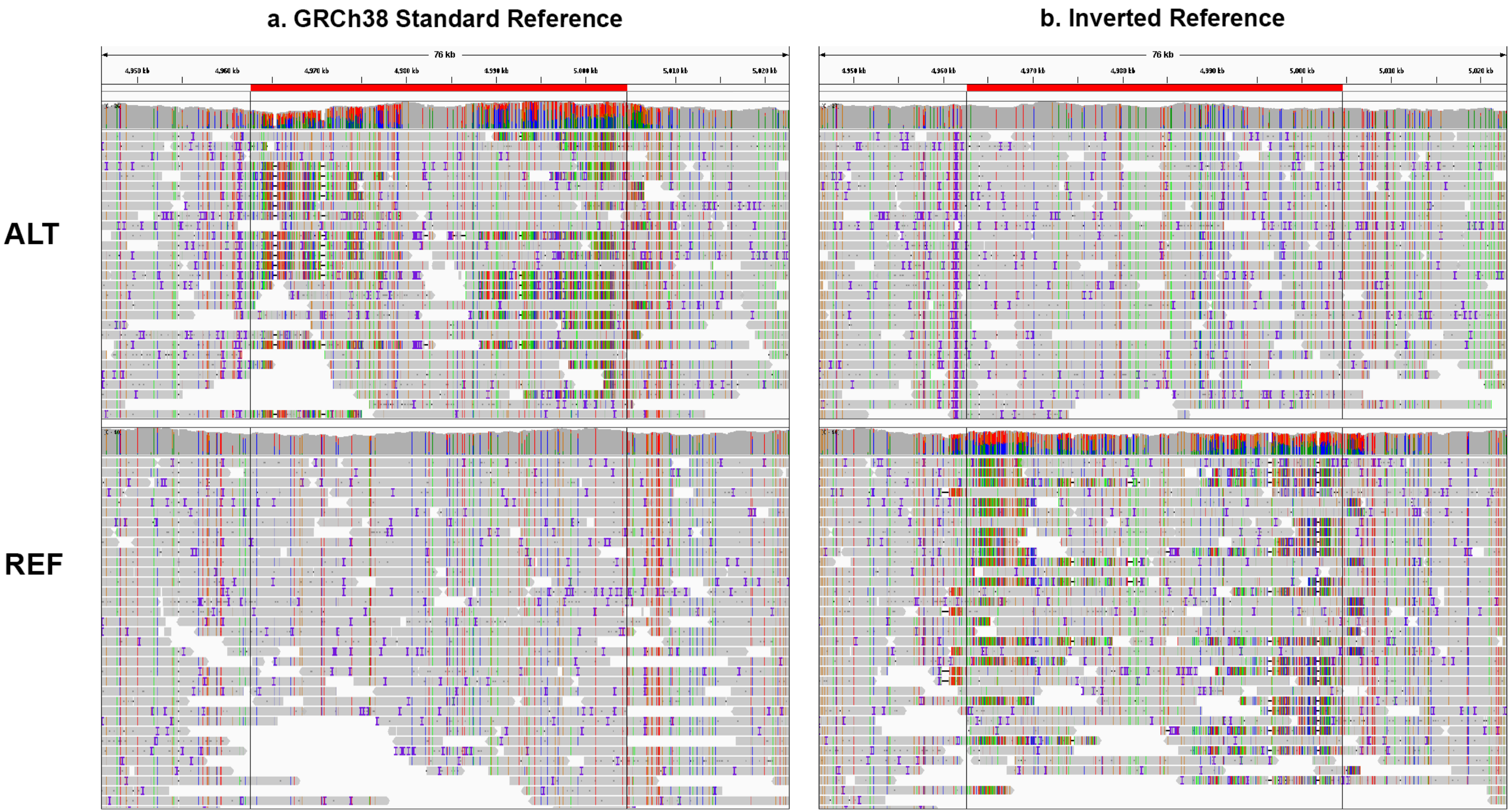

Fig. S1 The cleanly phased BAM files. In both panels, the upper alignments track is the ALT allele and the lower track is the REF allele. The inverted region is shown by the red bar. In panel a. the alleles are aligned to the GRCh38 consensus sequence; the ALT allele shows signs of a large inversion while the REF allele fits well. In panel b. the reads are aligned to the new reference, where the region between the proposed breakpoints is inverted; now the ALT allele fits well while the REF allele shows the same signs of a large inversion but with the pattern reversed.

Raw reads mapped to the assembled ALT allele

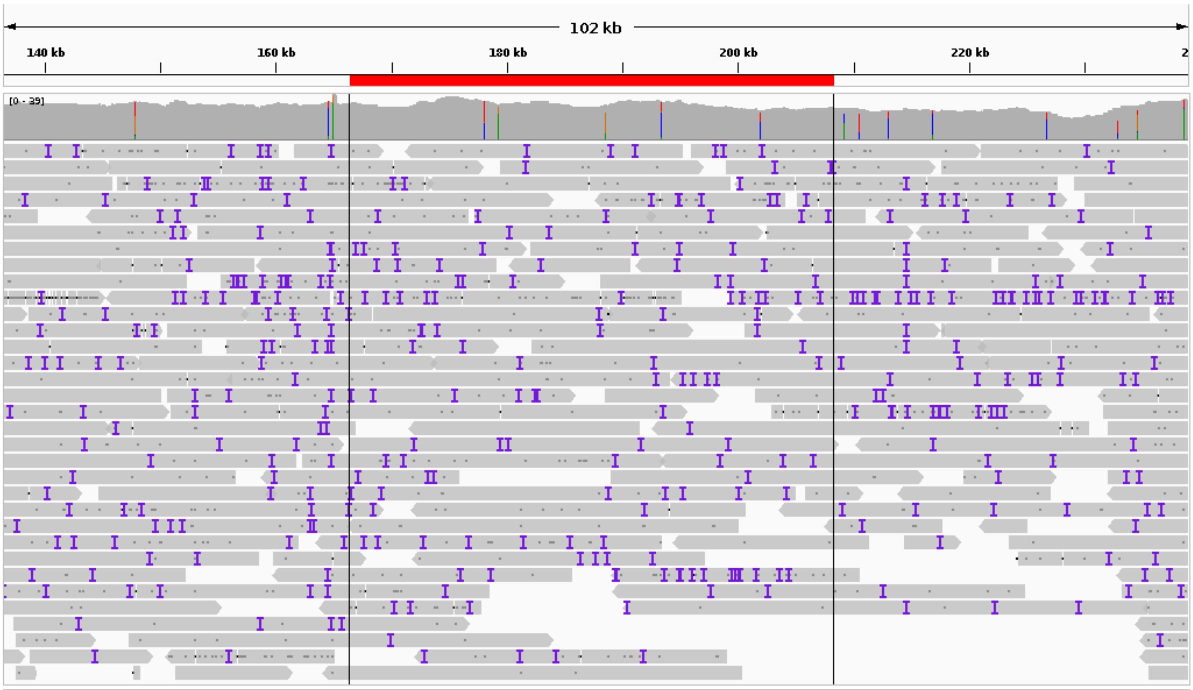

Fig. S2 The raw reads from the phased ALT allele mapped to the assembled ALT allele. INDELs of ≤5 bp are hidden. There are very few mismatched bases and no loci where more than half of the reads have a mismatched base, showing that the assembly is accurate.
