# Supplementary material for: Long read sequencing characterises a novel structural variant, revealing underactive AKR1C1 with overactive AKR1C2 as a possible cause of severe chronic fatigue
Source: J Transl Med. 2023 Nov 17;21:825. doi: 10.1186/s12967-023-04711-5 (PMC10655400; doi:10.1186/s12967-023-04711-5)
Supplement: Supplementary file 4 — Additional file 4: Further explanation of the lower 17-OHP/17,20α-diOHP ratio in the patient’s first luteal sample. [file 12967_2023_4711_MOESM4_ESM.pdf]

Further explanation of the lower 17-OHP/17,20α-diOHP ratio in the patient's first luteal sample

The patient had responded poorly to standard treatments for central adrenal insufficiency; oral glucocorticoids were not adequately absorbed due to the gastrointestinal dysfunction, while a hydrocortisone pump appeared to partially suppress the renin-angiotensin-aldosterone axis and so exacerbated the renal salt loss. She is therefore, uniquely in our experience, treated with physiologic replacement of adrenocorticotropic hormone (ACTH), the pituitary hormone that induces cortisol production in the adrenal glands, rather than with direct glucocorticoid replacement. This means that the adrenals are still being stimulated to produce cortisol in the normal way. At the time of the first luteal sample, the ACTH dose had been temporarily increased to cover an increased need for cortisol due to a minor injury, meaning that more 17-hydroxyprogesterone (17-OHP) was being metabolised by 21-hydroxylase as part of the cortisol synthetic pathway (Fig. S3, below). This increased stimulation was reflected in a higher serum cortisol concentration in that sample, the level of which was consistent with the ACTH dose used on that day. As a result, less 17-OHP was available for 20α-reduction and so did not exceed the limited capacity of AKR1C1.

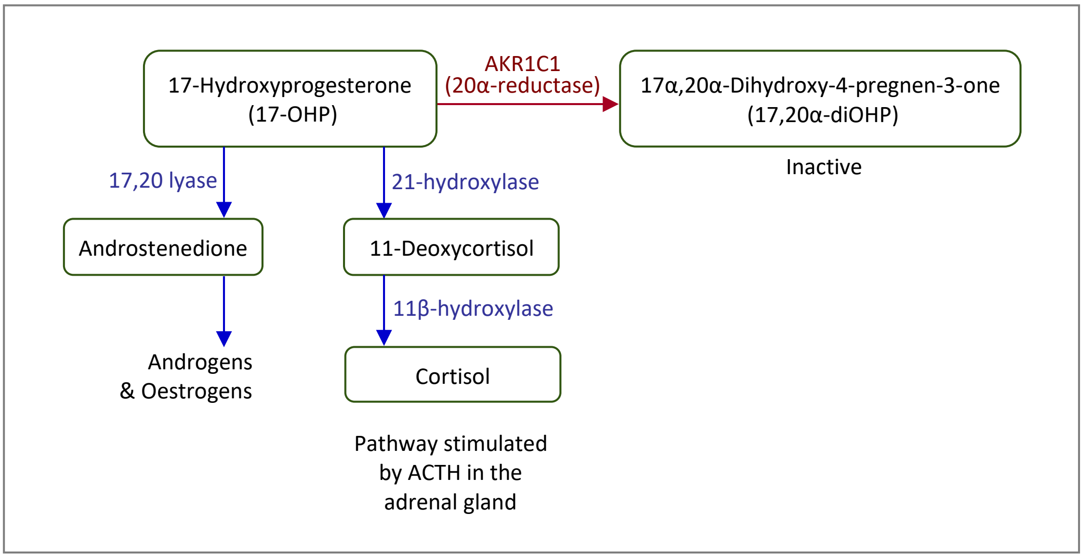

Fig. S3 Metabolism of 17-Hydroxyprogesterone. The 17,20 lyase pathway occurs in both the adrenals and gonads, while the cortisol pathway is exclusive to the adrenal cortex.
